# High Anion Gap Metabolic Acidosis and 5-Oxoprolinuria in a Hospital Setting Induced by Acetaminophen, Sepsis, and Malnutrition: A Case Report

**DOI:** 10.7759/cureus.75253

**Published:** 2024-12-07

**Authors:** Zainulabdeen Al-saedi, Mohammed A Miqdad, Lina Alatta, Bruce Spinowitz, Sheng Kuo

**Affiliations:** 1 Medicine/Nephrology, NewYork-Presbyterian Queens, New York, USA; 2 Research, Michigan State University, East Lansing, USA; 3 Nephrology, NewYork-Presbyterian Queens, New York, USA

**Keywords:** 5-oxoproline acidosis, 5-oxoprolinuria, acetaminophen, case report, high anion gap metabolic acidosis, malnutrition, pyroglutamic acidosis, sepsis

## Abstract

High anion gap metabolic acidosis (HAGMA) is a common biochemical abnormality in hospitalized patients, often linked to conditions such as lactic acidosis, renal failure, or drug toxicity. A rare etiology, 5-oxoprolinuria, resulting from acetaminophen use, malnutrition, and sepsis, is increasingly recognized in critically ill patients.

We report a 29-year-old male with a history of intellectual disability and normal baseline kidney function who was admitted with acute necrotizing pancreatitis and developed severe metabolic acidosis and acute kidney injury (AKI). Despite extensive management, including continuous renal replacement therapy (CRRT) and hemodialysis, he exhibited persistent HAGMA resistant to standard treatments. Following a prolonged hospital course complicated by various interventions, elevated urine 5-oxoproline levels were identified, leading to the discontinuation of acetaminophen and the initiation of N-acetylcysteine therapy.

This case highlights the challenges in diagnosing 5-oxoproline acidosis, particularly in the context of multi-factorial illness involving sepsis and malnutrition. The significant accumulation of 5-oxoproline underscores the metabolic stress associated with reactive oxygen species (ROS) depletion in critically ill patients. The recognition of this condition is crucial, as it indicates underlying metabolic derangements and necessitates prompt therapeutic intervention. Continued awareness and understanding of 5-oxoproline acidosis may improve outcomes in similar patients by guiding appropriate management strategies.

## Introduction

Metabolic acidosis is a prevalent biochemical abnormality in hospitalized patients and is generally classified as having a high or normal anion gap. High anion gap metabolic acidosis (HAGMA) is commonly encountered in hospitalized patients and is usually associated with a broad differential diagnosis, including lactic acidosis, renal failure, ketoacidosis, and drug toxicities [1]. However, less common etiologies, such as the accumulation of D-lactate or pyroglutamic acid (PGA) (5-oxoproline), should be suspected in cases without any other explanation. The latter has been primarily reported in the presence of chronic acetaminophen use. 5-oxoproline is an endogenous organic acid and a metabolite in the gamma-glutamyl cycle, involved in glutathione metabolism [2]. HAGMA can rarely be attributed to 5-oxoproline buildup, triggered by factors including prolonged acetaminophen use, nutritional deficiencies, and sepsis [2].

## Case presentation

A 29-year-old male with a past medical history of intellectual disability and normal baseline kidney function (creatinine 0.87 mg/dL), who was admitted for acute necrotizing pancreatitis, presumed secondary to valproate after exclusion of other causes, initially presented to the Emergency Department with vomiting. He was hemodynamically unstable, with hypotension (80/64 mmHg), tachycardia (120 BPM), tachypnea (28 RR), and oxygen saturation of 88%, along with severe electrolyte abnormalities and metabolic acidosis. On admission, creatinine was 3.76 mg/dL. He required IV fluids for resuscitation, endotracheal intubation, and ICU admission for ventilatory support, hemodynamic monitoring, and further management. The nephrology service was consulted for acute kidney injury (AKI) and continuous renal replacement therapy (CRRT).

In the ICU, he required IV fluids, vasopressors for blood pressure support, and IV sodium bicarbonate (NaHCO_3_) infusion for continued acidemia secondary to lactic acidosis and renal failure. On the second hospital day, CRRT was initiated for AKI and acidosis. He underwent surgical interventions for abdominal compartment syndrome, necrotizing pancreatitis, and feeding tube placement. CRRT was interrupted secondary to surgical procedures and clotting issues; this interruption phase included a failed trial of diuresis. However, CRRT was re-initiated without interruption on the 12th hospital day. On the 26th hospital day, he was switched to intermittent hemodialysis three times per week and transferred to the general medical floor.

CRRT and later hemodialysis temporarily closed the anion gap and increased bicarbonate levels, as shown in Tables 1-2. Hemodialysis was discontinued on the 32nd hospital day with increasing urine output and improving creatinine; however, he continued to have HAGMA with significantly low bicarbonate and unmeasured anions, resistant to high-dose per os (PO) bicarbonate supplementation. The hospital course was complicated by the interruption of feeding due to the displacement of the feeding tubes.

**Table 1:** BMP variables during different dates of hospital stay BMP: Basic metabolic panel; Na: Sodium; K: Potassium; Cl: Chloride; HCO_3_: Bicarbonate; BUN: Blood urea nitrogen; Cr: Creatinine; AG: Anion gap

BMP	Day 0	Day 5	Day 7	Day 15	Day 16	Day 28	Day 29	Day 35	Day 41	Day 47
Na (mmol/L)	133	135	135	133	134	133	136	135	141	145
K (mmol/L)	6.5	4.6	5.2	4.8	4	3.8	3.8	4.1	3.8	4.3
Cl (mmol/L)	91	101	101	98	101	94	92	95	98	101
HCO3 (mmol/L)	12	19	12	16	21	13	9	14	10	13
BUN (mg/dL)	39.1	18.7	26.2	25	13.8	45.2	17.1	34.2	70.2	40.1
Cr (mg/dL)	3.76	1.46	2.19	2.59	1.42	3.24	2.07	3.42	2.64	0.97
AG	30	15	22	19	12	26	35	26	33	31
Glucose (mg/dL)	368	230	175	177	156	222	226	137	180	202

**Table 2:** ABG analysis during different dates of hospital stay ABG: Arterial blood gas; PO_2_: Partial pressure of O_2_; PCO_2_: Partial pressure of CO_2_; HCO_3_: Bicarbonate

ABG	Day 0	Day 5	Day 7	Day 15	Day 16	Day 23	Day 34	Day 40	Day 42
PH	7.25	7.41	7.30	7.32	7.31	7.38	7.39	7.34	7.22
PO2 (mmHg)	82.0	87	94.5	89.4	174	142	112	23.3	23.9
PCO2 (mmHg)	36.5	37.8	34.8	35.9	36.4	35.2	30.5	107	66.5
HCO3 (mmol/L)	16.1	24	17.1	18.7	18.6	20.9	18.9	12.5	9.9

There was no history of salicylate, methanol, ethanol, isopropanol, or ethylene glycol use before or during the hospitalization. Serum lactate and beta-hydroxybutyrate levels were normalized on the third hospital day, as shown in Tables 3-4. The ventilator settings were appropriate, as shown in the blood gases (Table 2). However, he had been receiving 3-4 grams of acetaminophen daily from admission to the 34th hospital day for intermittent high-grade fever (38-39°C).

**Table 3:** Serum lactate level (reference range 0.5-1.6 mmol/L)

	Day 0	Day 5	Day 9	Day 15	Day 16	Day 25	Day 26	Day 33	Day 34	Day 40
Lactate	4.3	0.9	1.2	1.6	0.7	0.9	0.7	1.1	0.7	0.7

**Table 4:** Beta-hydroxybutyrate level (reference range 0.02-0.27 mmol/L) BHB: Beta-hydroxybutyrate

	12/27/23	12/31/23	1/3/24	1/26/24	1/26/24	1/27/24	1/30/24	2/5/24	2/6/24	2/11/24
BHB	0.29	5.56	0.19	3.52	0.10	0.08	0.09	0.11	0.11	0.07

5-oxoproline acidosis was suspected, and urine 5-oxoproline, along with D-lactate levels in the context of recent surgical intervention, were ordered. Acetaminophen was immediately discontinued. The urine 5-oxoproline level result was 1550 mmol/mol creatinine (reference range < 62 mmol/mol), and plasma D-lactate was 0.01 mmol/L (reference range 0-0.25 mmol/L). N-acetylcysteine 20-hour protocol (loading: 200 mg/kg IV for four hours, and maintenance: 100 mg/kg IV for 16 hours) was given, which initially improved the bicarbonate level. A serum 5-oxoproline level was requested, but it was not performed. The repeated urine 5-oxoproline level result was 2836 mmol/mol creatinine (reference range < 62 mmol/mol).

## Discussion

This was a very unusual case of acid-base disturbance in which secondary lactic acidosis, ketoacidosis, and uremic acidosis briefly complicated the clinical course. Despite a high dose of oral NaHCO_3_, CRRT, and hemodialysis, HAGMA persisted during the clinical course.

5-oxoproline acidosis is a distinct metabolic disorder, historically viewed as a rare genetic anomaly in the gamma-glutamyl cycle. However, it is now recognized as a significant cause of high-anion-gap metabolic acidosis in critically ill patients with malnutrition and acetaminophen exposure. This disorder's significance extends beyond acidemia, as it indicates severe metabolic distress triggered by elevated reactive oxygen species (ROS) levels, resulting from glutathione depletion [1].

5-oxoproline typically results from glutathione deficiency, serving as a crucial intermediate in the gamma-glutamyl pathway. This metabolic cycle generates glutathione, facilitates amino acid transport into cells, and regulates enzyme activity. Normally, sufficient glutathione stores suppress the enzyme gamma-glutamylcysteine synthetase, controlling cycle activity. However, when glutathione levels drop, 5-oxoproline accumulation disrupts this delicate balance, signaling potential metabolic disturbances [3].

Various medications can lead to PGA accumulation, with 5-oxoproline acidosis linked to both chronic and acute acetaminophen exposure. Acetaminophen's reactive metabolite, N-acetyl-p-benzoquinoneimine (NAPQI), depletes glutathione, disrupting feedback inhibition of gamma-glutamyl cycle synthase and causing PGA buildup. Additionally, drugs like flucloxacillin and vigabatrin inhibit 5-oxoprolinase, preventing PGA conversion to glutamate and contributing to its accumulation [1,4].

NAPQI is a harmful metabolite of acetaminophen that typically causes liver damage in overdose situations. However, at normal therapeutic levels, intracellular glutathione neutralizes NAPQI, which is then excreted via urine. Patients with severe kidney impairment, characterized by minimal urine production, are more susceptible to toxic acetaminophen metabolite accumulation, even at recommended doses. While hemodialysis rapidly reduces acetaminophen levels, it does not prevent further NAPQI production. Current research indicates that NAPQI itself is not effectively dialyzable, highlighting the importance of careful acetaminophen administration in cases of renal impairment [5].

High 5-oxoproline levels stem from diverse factors beyond genetic predisposition, leading to 5-oxoprolinemia. The relationship between symptoms and acidosis is unclear, suggesting that oxoproline itself may contribute to symptoms. Treatment involves addressing underlying causes, considering bicarbonate infusion for severe acidosis (pH < 7.0), and potentially administering acetylcysteine to replenish glutathione levels via cysteine supplementation, offering a multifaceted approach to managing this complex condition [6].

Patients with sepsis-related 5-oxoproline acidosis face primary risk from tissue damage caused by ROS. Effective management requires addressing sepsis directly, discontinuing potentially causative drugs, and considering NaHCO_3_ treatment [1].

N-acetylcysteine is commonly administered for acetaminophen intoxication, despite limited supportive studies, due to its theoretical benefits and minimal adverse effects. It restores hepatic glutathione levels, enhances the negative feedback of gamma-glutamylcysteine synthetase to reduce PGA generation, and replenishes glutathione stores as a precursor for synthesis. Additionally, N-acetylcysteine exhibits antioxidant properties and requires concurrent treatment of underlying conditions, such as infections. Refractory cases may necessitate hemodialysis to remove acetaminophen from the blood [7-9].

Additionally, nutritional support is vital, as malnutrition increases risk, and riboflavin deficiency may play a significant role in the development of 5-oxoproline acidosis, emphasizing the importance of comprehensive care [1].

Hemodialysis is initiated for markedly elevated serum acetaminophen concentrations or severe metabolic acidosis [10,11]. This treatment effectively clears 5-oxoproline and resolves metabolic acidosis [12]. Furthermore, renal replacement therapy (RRT), encompassing CRRT and hemodialysis, accelerates 5-oxoproline elimination [13,14]. RRT plays a vital role by removing excess PGA and metabolites, correcting severe metabolic acidosis, replenishing glutathione levels, and supporting renal function, highlighting its essential benefits in managing pyroglutamic acidosis [1,15].

## Conclusions

This case highlights the critical need to consider 5-oxoprolinuria as a potential etiology of HAGMA in critically ill patients, particularly those with concurrent factors such as malnutrition and sepsis. Despite aggressive management, including RRT, the persistence of metabolic acidosis underscored the importance of thorough diagnostic evaluation in similar clinical scenarios. The identification of elevated urine 5-oxoproline levels, linked to chronic acetaminophen use, serves as a reminder of the complex interplay between medications and metabolic pathways, emphasizing the necessity for vigilant monitoring in hospitalized patients. This case not only contributes to the understanding of 5-oxoprolinuria in a clinical context but also underscores the importance of integrating nutritional support and careful medication management in critically ill populations.
